# Prayer

**Published:** 1889-11-16

**Authors:** 


### Words of Consolation.

### PRAYER.

(*For Reading to the Sick.*)

A very real test of our sincerity in the spiritual life is prayer. We may fancy that we love God and delight in His Word, that we are submissive to His will, and fear His displeasure, but unless our delight is in His commandments, and we are ready at all times to ask His help, to praise Him, and to return thanks for His mercies, we are but half-hearted in our devotion to Him. The habit of communion and intercession with God should be constant. For who can expect to receive benefits without troubling himself to ask for them?

Ask and it shall be given you, saith our Lord, and in connection with these words cites the case of the neighbour who would not rise from his bed to give his friend three loaves until he had been worried by his importunity, and of the poor widow whose cause was avenged by the unjust judge for a like reason. "And shall not God avenge the cause of His elect which cry unto Him day and night, though He bear long with them? I tell you He will," saith Christ, "avenge them speedily." "With all supplication let your requests be made known unto God. Pray without ceasing," are St. Paul's counsels to the Church. By this, however, it is not meant that the Christian should be always on his knees, but that the general attitude of his mind should be one of dependence on and constant intercourse with God. There are seasons when a prolonged time of prayer and praise is our duty, and should be our pleasure. The Psalmist exclaims with rapture "at evening and morning and at noonday will I praise Thee because of Thy righteous judgments," and, again, "seven times a day will I praise Thee." These are instances of the devotions of the saints; and our Lord Himself, we know, spent whole nights in prayer to God. By them we learn the necessity of keeping our souls in close touch with the Almighty, and if we have neither time nor faith to imitate them we can by earnest and brief thought send up, as it were, arrows of prayer which will pierce through the gates of heaven and show our sense of God's unfailing help. All who are lying on a sick bed can seize the leisure and the opportunity given them to make these truths their own, and feel what bliss it is to have a Father who knows all we need, and will grant all to His faithful, loving children who come to Him for help. Ask, then, with humility and faith, in sorrow and distress, in pain and grief, the great God will lend a gracious ear to your requests if you plead in the name of His only and dear Son our blessed Lord and Saviour Jesus Christ.

